# Breastmilk Cell and Fat Contents Respond Similarly to Removal of Breastmilk by the Infant

**DOI:** 10.1371/journal.pone.0078232

**Published:** 2013-11-06

**Authors:** Foteini Hassiotou, Anna R. Hepworth, Tracey M. Williams, Alecia-Jane Twigger, Sharon Perrella, Ching Tat Lai, Luis Filgueira, Donna T. Geddes, Peter E. Hartmann

**Affiliations:** 1 School of Chemistry and Biochemistry, Faculty of Science, The University of Western Australia, Perth, Western Australia, Australia; 2 School of Anatomy, Physiology and Human Biology, Faculty of Science, The University of Western Australia, Perth, Western Australia, Australia; 3 Anatomy Unit, Department of Medicine, University of Fribourg, Fribourg, Switzerland; Bascom Palmer Eye Institute, University of Miami School of Medicine, United States of America

## Abstract

Large inter- and intra-individual variations exist in breastmilk composition, yet factors associated with these variations in the short-term are not well understood. In this study, the effects of breastfeeding on breastmilk cellular and biochemical content were examined. Serial breastmilk samples (∼5 mL) were collected from both breasts of breastfeeding women before and immediately after the first morning breastfeed, and then at 30-minute intervals for up to 3 hours post-feed on 2–4 mornings per participant. The infant fed from one breast only at each feed. Effects of pump versus hand expression for samples were evaluated. A consistent response pattern of breastmilk cell and fat contents to breastmilk removal was observed. Maximum fat and cell levels were obtained 30 minutes post-feed (P<0.01), with up to 8-fold increase in fat and 12-fold increase in cell content compared to the pre-feed values, and then they gradually decreased. Breastmilk cell viability and protein concentration did not change with feeding (P>0.05), although large intra-individual variability was noted for protein. Expression mode for samples did not influence breastmilk composition (P>0.05). It is concluded that breastmilk fat content, and thus breast fullness, is closely associated with breastmilk cell content. This will now form the basis for standardization of sampling protocols in lactation studies and investigation of the mechanisms of milk synthesis and cell movement into breastmilk. Moreover, these findings generate new avenues for clinical interventions exploring growth and survival benefits conferred to preterm infants by providing the highest in fat and cells milk obtained at 30 min post-expression.

## Introduction

Breastmilk composition is dynamic and variable among women [1]–[6]. Variations within a woman are less well understood, with breastmilk fat content having been shown to be influenced by breastmilk removal during either breastfeeding or breast expression in the short-term. Changes in other breastmilk components, such as protein and cell content, with feeding are not well understood, which may have contributed to the lack of sample standardization in previous studies, and thus the large variability in these breastmilk components reported both amongst and within women [3] [7], [8]. This highlights the need to establish how breastfeeding and breastmilk removal influence breastmilk composition and the mechanisms through which these changes are exerted. This is clinically important in the optimization of preterm infant nutrition, and the facilitation of the growth and survival of these susceptible infants.

Fat is a major digestible component comprising over 50% of the energy of breastmilk. It is highly variable and known to increase from the beginning to the end of a breastfeed or expression [2], [3], [9], [10]. Feeding-related factors, such as the amount of milk removed at the feed and feeding frequency, have been suggested to influence the fat content of milk. Daly et al. (1993) [11] however provided evidence that breast fullness or the volume of milk stored in the breast at any point of time was closely related to milk fat content (r^2^ = 0.68) in that fuller breasts had a lower fat content than less full breasts. In addition, the increase in milk fat content as the breast empties has been measured in women and other mammals, and several mechanisms for this change have been proposed [12]. One theory suggests that milk fat globules adsorb to alveolar membranes and are displaced only when the gland is near empty [12]–[14]. Another theory proposes a gradual filtration of the duct wall-adhering fat globules during breast emptying [15].

To further elucidate changes in milk fat and other components with the degree of breast fullness and illuminate potential mechanisms of feeding-dependent changes, we measured milk fat content pre-feed and at regular intervals post-feed during a three-hour period prior to the next feed. Simultaneously, the protein (soluble factor) and the cellular (non-soluble factor) contents of breastmilk were also measured. We sought to further investigate these associations by comparative analyses of milk biochemical and cellular composition and how these are influenced by milk removal by the infant. For this, we extended the previously studied time points of milk composition from immediately pre- and post-feed to 3 hours post-feed, and examined both the feeding and non-feeding breasts simultaneously. These relationships and responses to breast fullness were investigated longitudinally for each participant and cross-sectionally to ensure their reproducibility.

## Materials and Methods

### Ethics statement

All procedures involving human subjects were approved by the Human Research Ethics Committee of The University of Western Australia. Written informed consent was obtained from all participating mothers.

### Study population

Healthy breastfeeding mothers (28–40 years old; 6–39 weeks of lactation) (N = 6) were recruited in Western Australia. Exclusion criteria included maternal or infant illness or medical condition, use of medication, incidence of mastitis or nipple pain within 4 weeks prior to the first collection day, perceived or actual low milk supply or over supply, and/or low infant weight gain.

### Study design

The study design is summarized in Table 1. Collection of consecutive breastmilk samples (time series) were carried out at the same time of day, the morning, and each participant repeated these collections on 2 (N = 2 participants) or 4 (N = 4 participants) occasions during consecutive weeks (N = 4) or over the course of 6 weeks (N = 1) or 20 weeks (N = 1). On each collection day, participants were asked to collect approximately 5 mL breastmilk from each breast immediately before and after the first morning feed, and then at 30 minutes, 1 hour, 1.5 hours, 2 hours, 2.5 hours and 3 hours post-feed. Participants did not breastfeed or express any breastmilk for at least 3 hours pre-feed in order to ensure that the breasts were full or near full at the beginning of the feed. Similarly, no breastfeeding or breastmilk expression occurred for 3 hours post-feed other than that required for the study. The baby fed from one breast only, which was designated as the “feeding breast”, while the other breast was the “control or non-feeding breast”. Both breasts were sampled during the above time points in a given morning, with few exceptions as indicated in Table 1. The infant's weight was recorded before and after the feed using electronic baby weigh scales (Medela AG, Baar, Switzerland; resolution 2 g, accuracy ±0.034%) to provide a measurement of the breastmilk volume consumed during the feed. Each participant provided 24–64 breastmilk samples, with a total of 280 breastmilk samples collected and analyzed. Breastmilk samples were expressed by participants aseptically either by hospital-grade electric pump (Medela AG, Switzerland) or hand expression, with only one expression mode employed on each collection day. Breastmilk samples were stored in sterile polypropylene vials at approximately 20°C in the dark until transportation to the laboratory within an hour of the final collection.

**Table 1:** Characteristics of breastmilk collections and 24-hour milk productions for each of the participating breastfeeding dyads.

Participants	Total repeats	Collection time points (weeks)^1^	Modes of breastmilk expression^2^	Breasts sampled^3^	Time points missing^4^	Total samples collected	24-hour milk production (g)
Dyad 1	4	12, 14, 17, 33	H, H, H, P	F, F, FC, FC	0	48	878.4 (367+512)^5^
Dyad 2	4	6, 7, 8, 9	P, H, P, H	F, FC, FC, FC	3 (3 hrs)	50	809.2 (302+507)
Dyad 3	4	6, 7, 8, 9	H, H, P, P	FC, FC, FC, FC	1 (3 hrs)	62	726.5 (314+412)
Dyad 4	4	7, 9, 11, 12	H, H, P, P	FC, FC, FC, FC	0	64	809.6 (397+412)
Dyad 5	2	12, 13	P, P	FC, FC	0	32	-
Dyad 6	2	38, 39	H, H	FC, F	0	24	499.6 (270+230)
*Study total*	*20*	*6*–*39*	*H: 11, P: 9*	*F: 20, C: 16*	*4 (3 hrs)*	*280*	*744.7* 6

1 Collection time points refer to a morning during each of the named weeks. Weeks refer to postpartum infant age.

2 Modes of breastmilk expression refer to hand expression (H) and pump expression (P), and correspond to the weeks of collection.

3 Breasts sampled refers to F: feeding and C: control (non-feeding) breast, and correspond to the weeks of collection.

4 Time points missing refers to the number of time points within each collection day not sampled by the participant. The specific time point(s) is/are indicated within parentheses.

5 In parentheses, the productions of the left and right breasts are shown as (left+right).

6 Average 24-hour milk production (N = 5).

### Breastmilk fat content

Breastmilk samples were hand-mixed well and 200-µL aliquots were frozen at −80°C. Fat content was measured in the fresh samples by the creamatocrit method [16] using Creamatocrit Plus (Medela, Inc, McHenry, Illinois). Creamatocrit measurements strongly correlate to the biochemical spectroscopic esterified fatty acid assay [10], [17].

### Breastmilk cellular content

Total cells were isolated from fresh breastmilk samples as described by Hassiotou et al. [18]. Briefly, breastmilk was diluted with equal volume of sterile Phosphate Buffered Saline (PBS, pH 7.4, Gibco, USA) and centrifuged at 805 *g* for 20 minutes at 20°C. The fat and liquid (skim milk) parts were removed and the cell pellet was washed three times in PBS. Total cell content and viability were determined with a Neubauer haemocytometer by Trypan Blue exclusion.

### Breastmilk protein content

Breastmilk protein content was determined in sample aliquots that had been previously frozen (−80°C) and thawed at 37°C prior to defatting. Protein was quantified in the skim milk fraction by the Bradford protein assay using a commercial protein reagent (Bio-Rad Laboratories, Richmond, California). Human milk protein standards were prepared as described by Atwood and Hartmann [12] and the assay was performed as described in Mitoulas et al. [10], with a 1 in 30 dilution of the skim milk samples, using an automatic liquid handler (Janus, PerkinElmer). The recovery of a known amount of protein added to the milk samples was 98±4% (N = 8). The detection limit of the assay was 0.012±0.003 g/L and the inter-assay CV was 8.6% (N = 14).

### Twenty-four hour milk production

Twenty-four hour milk production was determined using the test-weighing method [19], while degree of breast fullness was calculated by analysis of breastmilk fat content before and after each breastfeed, as described previously [2], [11]. Sampling and measurements for these procedures were performed over a period of 24–28 hours on days other than those of the morning feed sampling, with correction of measurements to 24 hours (Table 1).

### Statistical analysis

Statistical analyses were performed in R 2.9.01 [20] using the base packages and libraries nlme [21] and lattice [22] for linear mixed effects models and lattice plots, respectively. The number of samples and time sequences over time (N = 20) was adequate to describe consistent trends within and among mothers. Summary statistics are presented as mean ± SD to show variation, and estimates are presented as mean ± SEM.

Feed volume at the first morning feed of the 24-hour milk production (N = 5) was compared to feed volumes for the same participants at the experimental time series sessions (N = 17) using a linear mixed effects model with condition (24-hour/experimental) as the predictor and participant baseline as the random effect.

Patterns in milk fat, cell and protein contents and the percentage of viable cells were investigated using linear mixed effects models. Two random effects were considered: similar baseline levels for each individual and similar baseline levels for each breast, grouped by individual, thereby allowing for one breast to be significantly higher than the other for an individual, but that this difference not be consistent among women. Due to the very different patterns between the feeding and non-feeding breast, analyses were done for the whole data set as well as separately for the feeding and non-feeding data subsets. To compare values at different time points, the eight time points were considered as factors to allow for a non-linear pattern in the data. The 30-minute post-feeding time point, which showed the highest fat and cell contents, was used as a reference level for comparison with the other time points.

Significance of the differences was tested in several ways. To calculate an overall P value for the significance of a factor, analysis of variance (ANOVA) was used to determine an F statistic and associated P value. This tested the null hypothesis that all of the group means are the same. If a factor was significant, post-hoc testing was done to identify which groups differed from the reference. The relationship between fat and each of the other factors, differences between the feeding and non-feeding breasts, and between pump and hand expression were tested for by considering these as univariate predictors of the milk composition factors and by adding them to the time point models and testing for significance.

Data was missing for the final (3-hour) time point of four feeds as the infant fed before this time point; four control feeds (samples not collected); one 24-hour milk production, which the participant was unable to complete; one feeding sequence for fat content due to insufficient sample volumes; and two feeding sequences for protein concentration due to insufficient sample volumes. For the feeding sequences, it was not necessary to have identical length sequences, as the linear mixed effects model approach can encompass different sample times/numbers for different individuals. For all other analyses, complete case analysis was used.

## Results

### Breastfeeding influences milk cell and fat contents

The 24-hour milk productions of the study participants were all within the normal range of (500–878 g) (Table 1). The volume of milk removed during the examined morning feed ranged 58–156 mL, with a mean±SD of 108±32 mL (N = 19). This range was similar to the first morning feed of the 24-hour period (54–146 g), with no significant difference in feed volume between the experimental morning feed and the normal first morning feed of the 24-hour period for each mother (P = 0.96). These results suggest that the experimental conditions of the first morning feed were similar to those of the normal breastfeeding patterns of the examined dyads.

**Figure 1:**
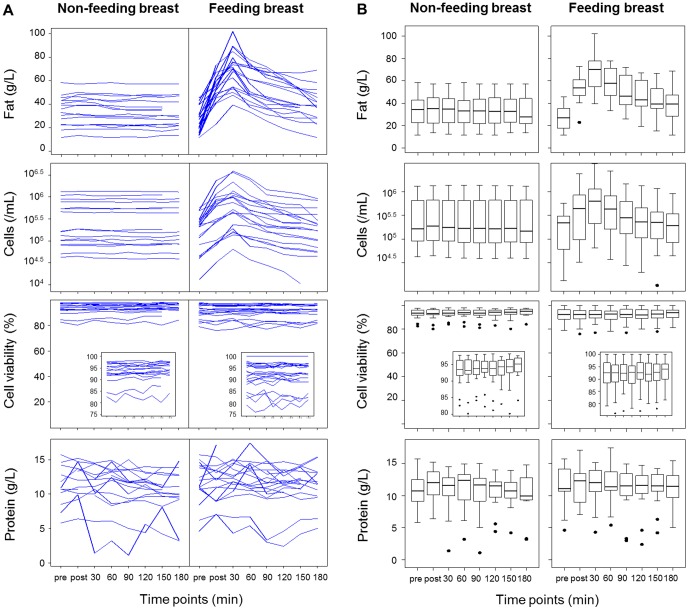
Responses of milk fat, cellular and protein contents and cell viability to feeding in the feeding and non-feeding (control) breasts. (A) Inter- and intra-individual variation in responses to feeding. Each line represents a feed, and all feeds from all participants are shown. (B) Distribution of values from all feeds and participants (N = 6) for the feeding (N = 20) and non-feeding (N = 16) breasts at each time point. Pre: immediately pre-feed collection; post: immediately post-feed collection. Boxes show first and third quartiles; horizontal bars within boxes indicate median values; and ‘whiskers’ plus outlier dots show the range of values.

In the feeding breast there were marked increases in both milk fat (P<0.001) and cell content (P<0.001) in response to milk removal (Fig. 1, Table 2). Following this initial increase, the highest levels were observed 30 minutes post-feed, after which milk fat and cell content gradually decreased as the breast synthesized milk (Fig. 1). The values obtained immediately post-feed and at 60 minutes were the closest to the maximum values observed at 30 minutes post-feed, but still significantly lower (P<0.01), whereas all other values were much lower (P<0.001). No significant temporal patterns were seen for protein content (P = 0.26) or cell viability (P = 0.96) in response to feeding (Fig. 1).

**Table 2:** Breastmilk composition among participants for the feeding (N = 20) and non-feeding (control) (N = 16) breasts.

Component	Feeding breast		Non-feeding breast	
	Range	Mean ± SD	Range	Mean ± SD
Fat content (g/L)	11.6 – 102.1	48.7±18.1 (N = 148)	11.6 – 58.4	33.5±12.4 (N = 124)
Pre-feed fat content (g/L)	11.6 – 45.9	27.2±11.3 (N = 19)	11.6 – 58.4	33.9±12.6 (N = 16)
Maximum fat content (g/L)	39.4 – 102.1	69.1±16.4 (N = 19)	13.4 – 58.4	35.2±12.9 (N = 16)
Cell content (/mL milk)	1.1×10^4^–3.9×10^6^	4.8×10^5^±6.0×10^5^(N = 156)	3.9×10^4^ – 1.4×10^6^	4.1×10^5^±4.0×10^5^ (N = 124)
Pre-feed cell content (/mL milk)	1.3×10^4^ – 5.7×10^5^	2.2×10^5^±1.6×10^5^ (N = 20)	4.3×10^4^ – 1.3×10^6^	4.1×10^5^±4.1×10^5^ (N = 16)
Maximum cell content (/mL milk)	6.5×10^4^ – 3.9×10^6^	1.0×10^6^±1.1×10^6^ (N = 20)	4.4×10^4^ – 1.4×10^6^	4.3×10^5^±4.3×10^5^ (N = 16)
Protein concentration (g/L)	2.4 – 17.4	11.4±2.9 (N = 134)	1.1 – 15.7	10.8±3.0 (N = 118)
Pre-feed protein content (g/L)	4.6 – 15.7	11.5±3.0 (N = 17)	5.8 – 15.7	10.9±2.7 (N = 15)
Maximum protein content (g/L)	7.0 – 17.4	13.2±2.9 (N = 17)	8.1 – 15.7	12.8±2.2 (N = 15)
Cell viability (%)	76.0 – 100.0	91.5±5.8 (N = 156)	80.2 – 98.2	93.2±4.2 (N = 124)

In contrast, in the control breast, neither fat (P = 1.00); protein (P = 0.08); cells (P = 1.00); nor cell viability (P = 0.77) of breastmilk changed significantly, with values remaining within narrow ranges relative to the values seen in the feeding breast (Fig. 1, Table 2). Within a breast and a feed, milk protein concentration was less consistent and more variable than the other measures of breastmilk composition (Fig. 1, Table 2).

**Figure 2:**
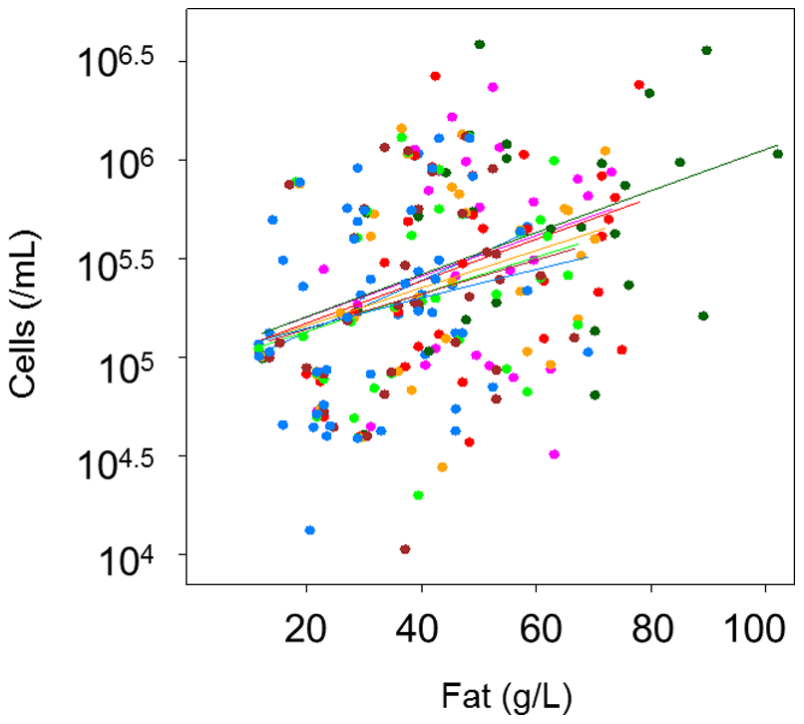
Relationship between milk fat and cell contents in all the participants for all the feeds and repeats (N = 36). Lines indicate different sampling points, showing a consistent pattern over time. Different colors indicate different sampling time points. Cell content was strongly and linearly related to fat content (P<0.001), after accounting for the effect of time.

**Figure 3:**
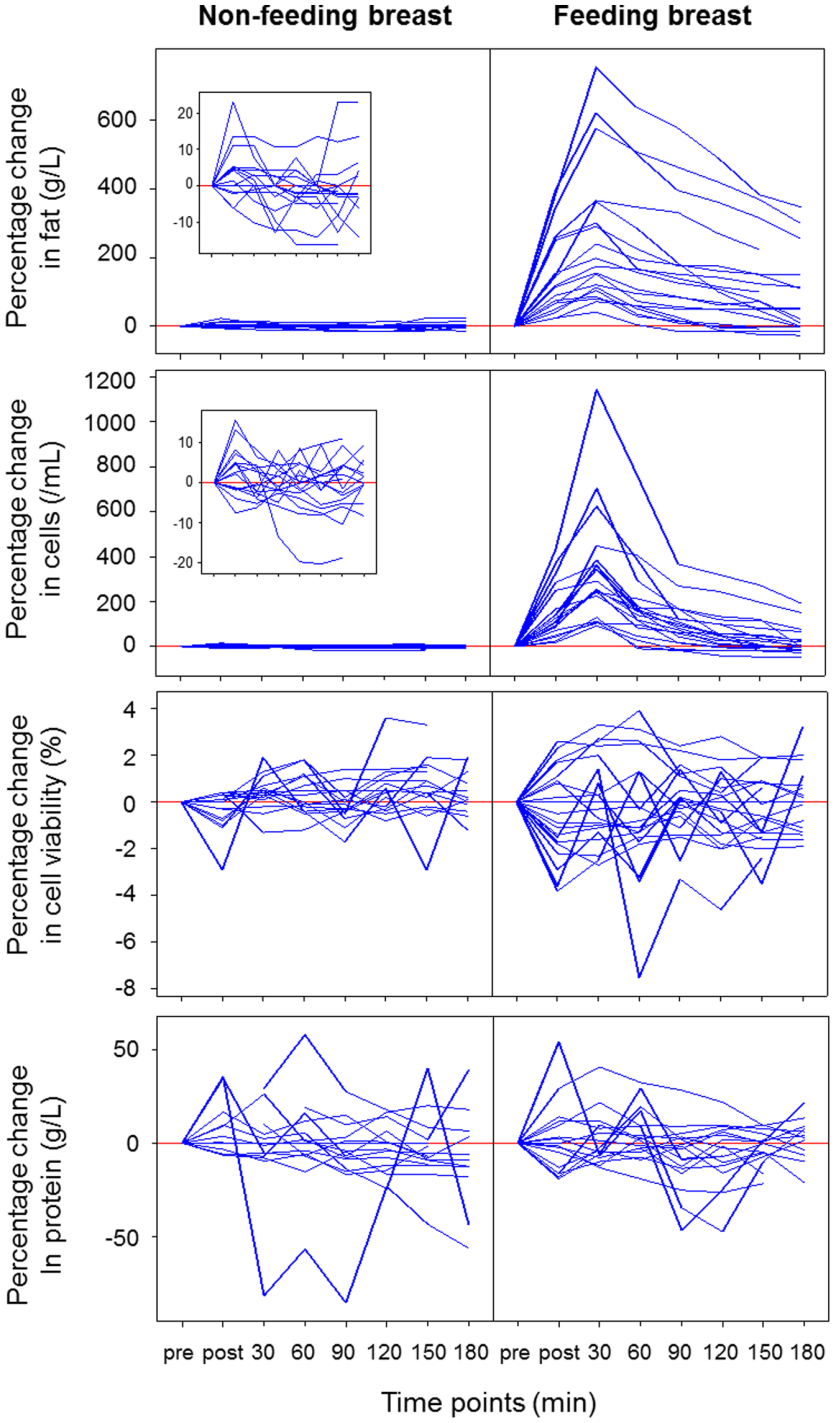
Percentage increase in the measured fat, cell and protein contents, and cell viability relative to the starting pre-feed values, calculated as (measured value – pre-feed value)/pre-feed value) ×100. Note that towards the end of the feed this can be negative, when the measured value goes below the pre-feed value. Pre: immediately pre-feed collection; post: immediately post-feed collection.

The relationship between fat content, and thus breast fullness, and milk cell content in the feeding breast over time (peaking at 30 minutes post-feed) was consistent in all participants (Figs. 1B, 2). Higher milk fat content was always associated with higher cell content (Fig. 2). In contrast, neither milk protein nor cell viability was related to fat content, and thus the degree of breast fullness (Fig. 3), with either no change or peaks at various time points post-feed. Consistent with the absolute values, the greatest percentage increase (relative to the pre-feed value) in milk fat and cell contents of the feeding breast occurred at the 30-minute time point (Fig. 3). No association was found between the percentage increase in milk fat or cell content either immediately or at 30 minutes post-feed and the volume of milk removed during the feed (P>0.05). However, the percentage increase in milk fat either immediately post-feed or at 30 minutes post-feed was strongly related to the pre-feed fat (P<0.001).

### Breastmilk composition varies both among women and between the breasts of a woman

**Figure 4:**
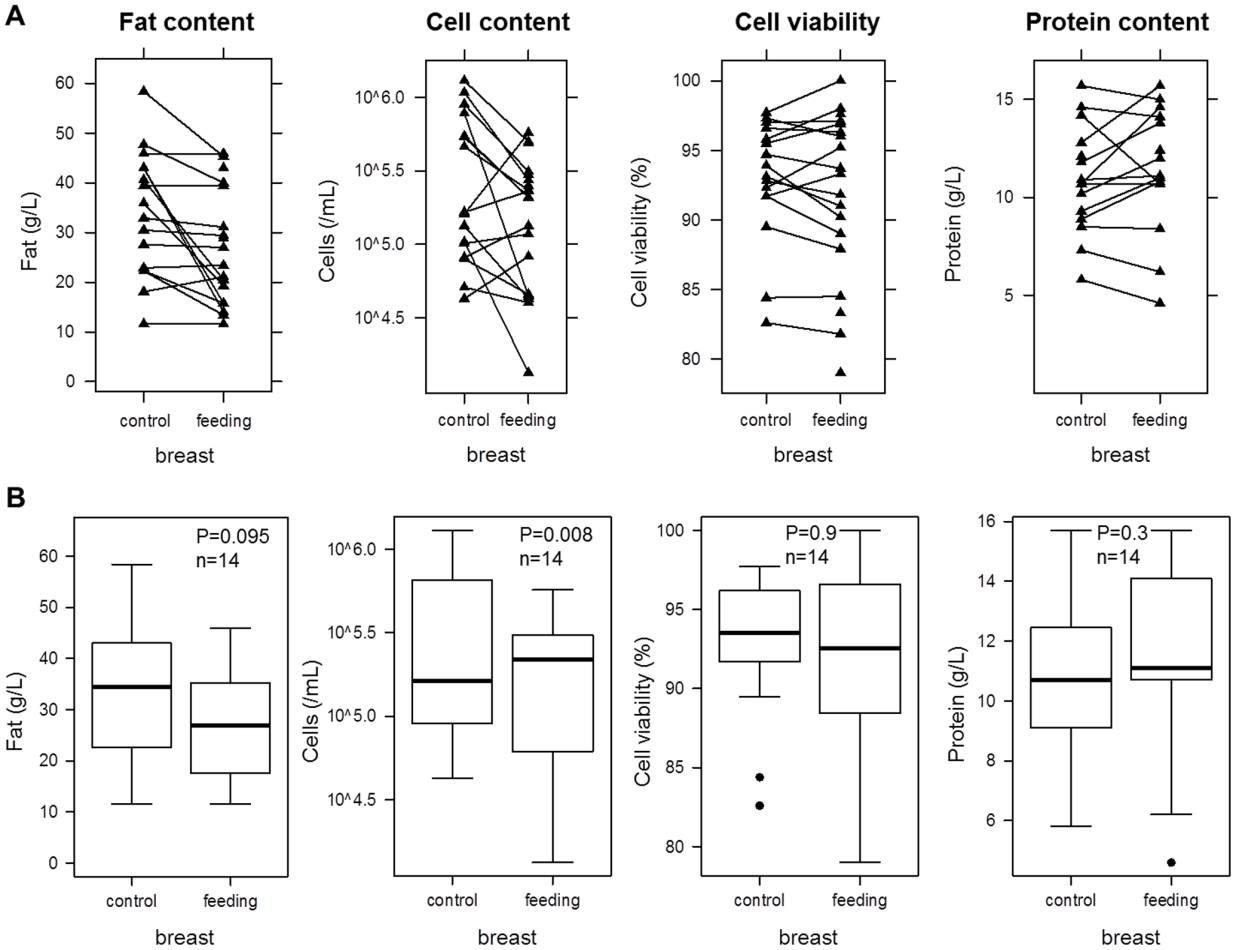
Comparison of pre-feed values between the feeding and the non-feeding (control) breasts for milk fat, cell and protein contents, and cell viability among participants and different feeds. (A) Direct comparison within each feed. (B) Distribution of pre-feed values for each variable and each breast.

**Figure 5:**
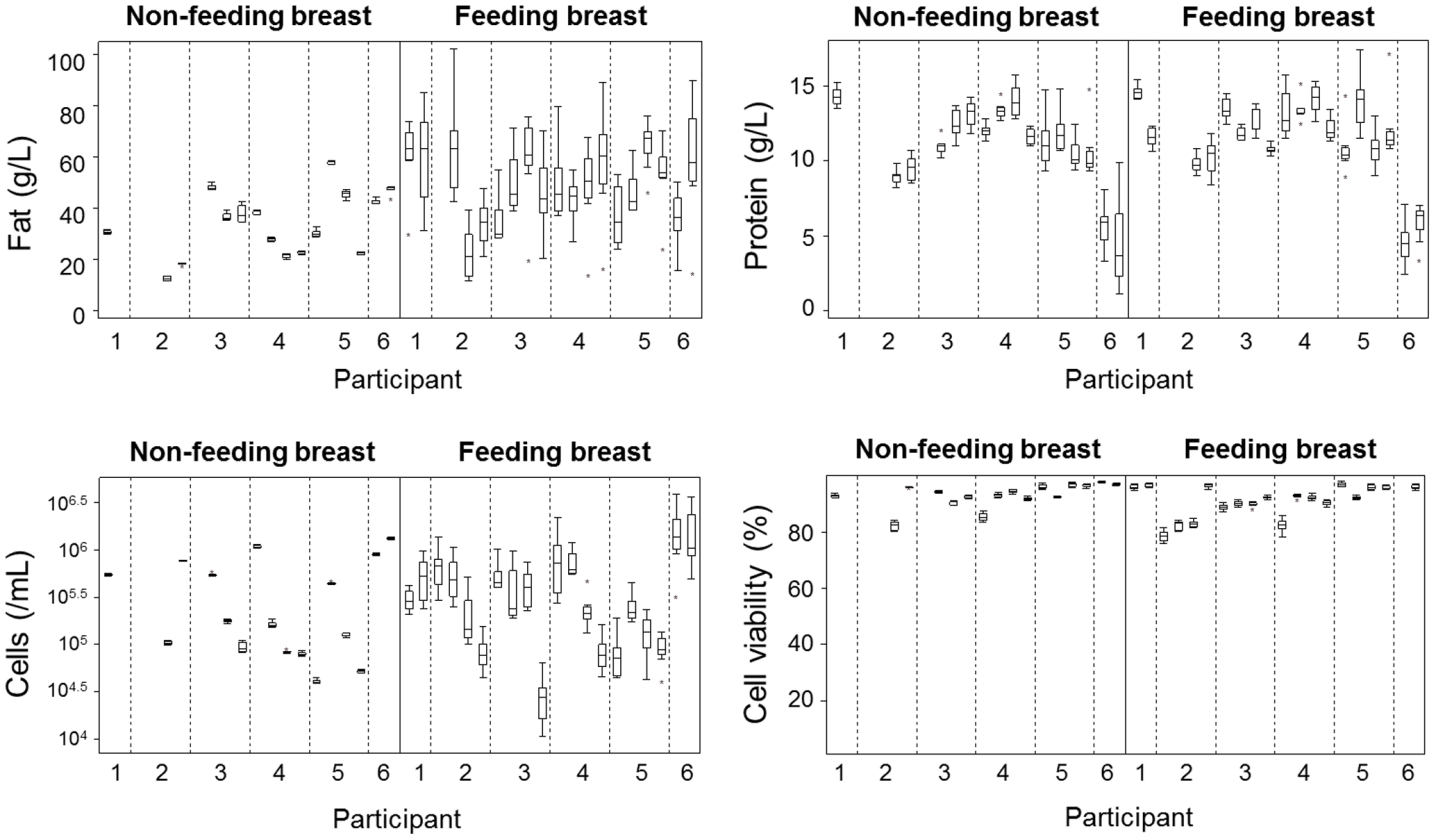
Inter- and intra-individual variation in milk fat, cell and protein contents and cell viability recorded. Variation between feeds and breasts within and among individuals. Values for control (non-feeding) and feeding breasts are on the left and right sides of the plots, respectively.

Breastmilk composition varied both among and within participants, both with day of study and between breasts. Some participants had significantly higher pre-feed cell and fat content in the control breast than in the feeding breast, suggesting that they started the sampling with a fuller feeding breast (Figs. 1A, 4). Individual differences were observed in breastmilk composition both in median values and in variability (Figs. 5, S1–S8 in File S1). For example, milk from participant P6 had consistently high cell viability and cell content, with the latter showing little variation (Fig. 5). By contrast, milk from participant P2 had variable cell viability, whereas participant P3 milk had the most variation in cell content (Fig. 5). Variation in milk protein concentration was similar within and among individuals. Significantly different patterns were seen in breastmilk composition between breasts (P<0.001). There were noticeable differences in fat and cell contents between the feeding and control breasts, with the control breast showing much less variation than the feeding breast (Fig. 5). In contrast, the inter-individual variation in cell viability was greater than the intra-individual variation (Fig. 5).

### Expression by either hand or breast pump yields similar breastmilk composition

**Figure 6:**
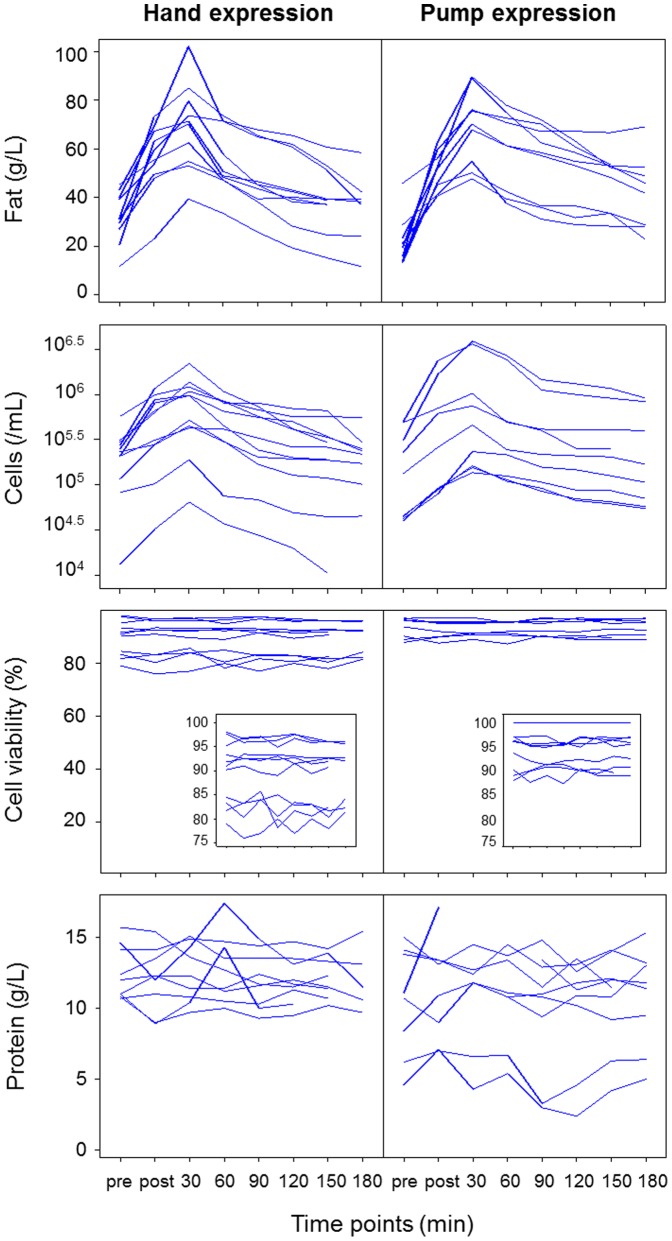
Effects of expression mode (hand versus pump expression) on milk fat, cell and protein contents, and cell viability for the feeding breast (N = 20 for cells and cell viability; N = 19 for fat; N = 18 for protein). Pre: immediately pre-feed collection; post: immediately post-feed collection.

In addition to feeding, we compared the expression of milk by hand with that of pumping with an electric breast pump in terms of breastmilk composition in the feeding breast (Figs. 6, S9–S12 in File S1). For milk cell content, no interaction was found between expression mode and the timing of breastmilk sample collection (P = 0.901), nor was there an effect of expression mode (P = 0.416) (Fig. 6). For milk fat content, while there was no effect of expression mode (P = 0.978), hand expression pre-feed was associated with lower milk fat content (P = 0.043). No effect of expression mode on milk protein concentration or cell viability was observed (P>0.05).

## Discussion

This study demonstrates that a strong association exists between the fat and cell content of breastmilk and an inverse relationship of both these parameters with the degree of breast fullness. Both fat and cell content increased from pre- to post-feed, peaking 30 minutes post-feed before gradually declining in the period prior to the next feed, likely reflecting short-term milk synthesis. How are breastmilk fat and cell contents related and why do they change with the degree of breast fullness? It was previously assumed that the highest fat content in breastmilk occurred immediately post-feed, which has been explained theoretically by the physics of milk fat globule flow through the mammary ducts. The newly discovered peak of breastmilk fat at 30 minutes post-feed and its close relationship with cell content suggests that other and/or additional mechanisms may be responsible for this phenomenon, and at the same time emphasize the importance of appropriately timed breastmilk collection when studying the effects of milk composition on the protection, nutrition and development of infants.

Breastfeeding stimulated a universal orchestrated change in milk fat and cell content over time (3 hours) in the feeding breast suggesting that this pattern is relatively conserved (Fig. 1). Higher levels of milk fat and cell content were observed immediately post-feed and maximum levels at 30 minutes post-feed, with 1.5- to 8-fold increase in fat and 2- to 12-fold increase in cell content compared to pre-feed values. Subsequently, breastmilk fat and cell levels gradually decreased over time. We suspect that the peak in breastmilk fat at 30 minutes post-feed may reflect a delay in the increase in milk synthesis, and therefore the 5–7 mL milk sampling at 30 minutes drained the feeding breast further, causing an additional increase in milk fat content. After 30 minutes, it appears that the rate of synthesis exceeded the sample volumes removed. In contrast to the feeding breast, milk fat and cell contents did not change in the control breast over time despite the expectation that fat content would decrease over time as the breast synthesized milk. Based on the 24-hour milk production rates of the control breasts (270–512 g), the calculated milk synthesis rates would be 5.6–10.7 g milk/30 minutes. Thus, removal of a 5–7 mL sample of milk every 30 minutes approximately matched the rate of milk synthesis in these breasts, and therefore the sampling procedure arrested the expected decline in milk fat content over time. These data suggest that the milk synthesis rates were higher in the feeding than the control breasts, which were not stimulated by removal of greater milk volumes. This agrees with previous studies showing that greater increases in fat content are associated with larger volumes of milk removed, and that milk synthesis rates of the left and right breasts may be discrepant and respond to feeding independently, with drained breasts synthesizing milk at rates higher than fuller breasts [4], [11]. This is consistent with the autocrine control of short-term milk synthesis. Moreover, it supports the practice of feeding at short intervals to allow breasts to maintain and/or increase milk synthesis rates, and thus milk supply. In terms of endeavoring to increase milk supply by draining the breast of milk as much as possible so that milk synthesis rates increase, it may be pertinent to express a small volume of milk 30 minutes after the pumping or feeding session. Further studies are required to investigate this.

The association between the fat and cell contents of milk and their maxima at 30 minutes post-feed pose questions as to the mechanisms by which these occur. The filtration theory proposed that while milk remains static in the breast the fat globules cluster and adhere to the walls of the alveoli and ducts, and are then filtered out as milk is gradually removed during either breastfeeding or breastmilk expression [15]. The adsorption theory suggested that fat globules remain adsorbed to the membranes of the alveolar cells by H-bonding and London dispersion forces interactions and are only displaced when the gland is near empty due to a decrease in the surface area available for this bonding [12]–[14]. Given that milk cells are also known to form clusters and have an overlapping range of diameter with milk fat globules in women [23], [24], both theories are rational explanations for the immediate post-feed increase in milk cell content. However, because they base their mechanistic explanations on the physicochemical phenomenon of milk removal from the breast, they can only explain the peak in milk fat and cell content at 30-minutes post-feed if it is assumed that milk fat globule and cell clusters that remain attached to the epithelium after the end of the feed are washed off into the milk during the next 30 minutes.

A more likely explanation for the 30-minute post-feed peak in fat content could involve both or either of the physical process of milk removal from the breast and the biology of milk synthesis in the lactocyte. The latter may involve feeding-stimulated timed synthesis and secretion of lipids and/or other milk components, such as proteins, which may coincide with changes in gene expression that allow active detachment of cells from the epithelium, hence the close relationship between breastmilk fat and cell content. To shed some light into this, we examined whether protein concentration changed pre- and post-feed. We did not observe a consistent pattern for protein that was similar to fat and cells, which is in accordance with previous studies [25]. We found strong inter-individual variations in protein responses to feeding that included patterns from no change to an increase at various time points post-feed. Since protein is a soluble factor in milk, these changes cannot be explained by the physicochemical phenomena proposed by either the adsorption or filtration theories. Therefore it is more likely that the changing breastmilk fat and cell contents in response to feeding are due to a biological response of the breast epithelium to milk removal. This process could be mediated by suckling-stimulated hormonal changes (e.g. in oxytocin or prolactin) and/or changes in gene expression that initiate active cell detachment from the epithelium, as we have previously shown [26] [9], and merits further investigation. It is of note that the changes in milk content with feeding can only be observed for particles of a minimum size, since they have not been observed for casein micelles [25]. Given the known size ranges for casein micelles (0.01–0.3 µm) [27], fat globules (<1–12 µm) [28], and milk cells (4–25 µm) [23], it can be postulated that very small fat globules may not participate in the changes in milk fat content with feeding.

It has been previously proposed that feeding-induced changes in breastmilk composition may be of physiological significance for the breastfed infant [9]. The post-feed increase in milk fat has been implicated in short-term appetite control as well as the development of the infant's appetite control system [9]. However, the role of milk cells for the infant is not yet clear. Recently, we demonstrated the presence of a cellular hierarchy in breastmilk, from multipotent stem cells to progenitors to more differentiated mammary cells [26] [18]. It has also been shown that post-feed milk contains less α-lactalbumin mRNA than pre-feed milk, hence less or fewer differentiated cells [29]. Thus, it could be speculated that the increase in milk cells post-feed and the maximum of 30 minutes might be reflective of a change in breastmilk cellular composition, i.e. the proportion of the different cell populations. Indeed, expression of STRO-1, a cell surface protein, has been shown to decrease after the feed, with the lowest expression found at 1 hour post-feed, after which it gradually increases again [26]. Changes in expression of this or other molecules involved in cell adhesion may provide a mechanistic explanation for the changes in breastmilk cell content after a feed and the 30-minute post-feed maximum. Given that feeding-induced breastmilk cell compositional changes have only been explored in one mother so far due to breastmilk volume limitations, this merits further investigation to unravel the mechanisms mediating the response of the breast epithelium to feeding and the potential functions of the different milk cell populations for the infant.

Although the overall patterns of change in milk fat and cell contents with feeding were remarkably consistent among and within participants, inter- and intra-individual variations were observed in the absolute values of milk fat, cell, protein and cell viability (e.g. on different days or for different breasts). For cell viability, the inter-individual variation was greater than the intra-individual variation. It is noteworthy that cell viabilities were high (75–100%), which is in agreement with previous literature [30]. In some participants, pre-feed cell and fat levels were higher in the control breast than in the feeding breast, demonstrating that these individuals started the sampling with a fuller feeding breast [2]. This is likely to be a consequence of our study protocol that asked the participants to feed from the fullest breast. This, together with the similar response of milk fat and cells to milk removal, further supports an association between the cell content of breastmilk and breast fullness. Our pre-feed samples agree with some published estimates of cell content [23], [31], suggesting that sampling of fuller breasts has been typical in previous studies. Since the degree of fullness of the breast has not been systematically considered in sampling protocols, it can at least in part explain the large inter- and intra-individual variation in milk cell content reported in the literature [7], [23], [31]. Thus, this study highlights the importance of standardization of sampling protocols for studies of breastmilk composition, taking into account the degree of breast fullness, to avoid misinterpretations of findings and have a correct basis for comparisons between studies. In addition to variations between breasts and with the degree of breast fullness, significant differences were found between the curves for the different days within individuals. This intra-individual variation suggests that one feed is not representative of an individual. Factors influencing this may be related to the stage of lactation, with milk cell content being typically higher in the early weeks of lactation. Differences were less pronounced for milk fat content and cell viability. The above further reinforce the importance of standardization of sampling protocols and the need for further studies to delineate factors other than the degree of breast fullness that influence breastmilk composition.

The mode of milk expression for the sampling did not relate to breastmilk composition, with the exception of the fat content of the pre-feed breastmilk sample, which was found to be lower with hand expression compared with pumping. Pumping is likely to provide a more homogeneous sample from the breast due to the application of a standardized vacuum selected by the mother for the duration of the session. This borderline effect could reflect differences in hand expression technique and may be further explored in more systematic sampling protocols with standardization of the hand expressing technique.

In summary, we show a close association between milk fat and cell contents, and how they change with the degree of breast fullness. We demonstrate that the highest fat and cell contents of milk can be obtained within 30 min post-feed, and these findings can be used to standardize and optimize sampling protocols in lactation studies. Our findings give new insight into the variation in milk fat and cell contents and their response to breastfeeding, suggesting that both milk fat and cell contents at any given time are related to the degree of breast fullness and potentially the rate of milk synthesis of the breast. Importantly, these findings have clinical potential for increasing the caloric content of milk expressed by mothers of preterm infants, thus facilitating the growth of these infants. Further investigations are needed to elucidate regulation of breastmilk synthesis as well as potential mechanisms involved in the movement of cells and fat into breastmilk. Moreover, this study generates new avenues for examining the impact of a clinical intervention involving provision to preterm infants of the highest in fat and cells milk obtained at 30-min post-expression.

## Supporting Information

File S1 **Inter- and intra-individual variation in breastmilk composition by study week, breast type (right or left), and expression type (pump or hand expression). File contains the following figures:** **Figure S1.** Inter- and intra-individual variation in breastmilk fat content by study week. The top row shows the feeding breast, whereas the bottom row shows the non-feeding (control) breast. Each repeat/week is indicated with a different line pattern (week one – solid line; week two ‘—’, week three ‘….’, week four ‘-.-.-.’). Each column represents one participant (N = 6). Pre: sample collected immediately pre-feeding; post: sample collected immediately post-feeding. **Figure S2.** Inter- and intra-individual variation in breastmilk cell content (counts/mL milk) by study week. The top row shows the feeding breast, whereas the bottom row shows the non-feeding (control) breast. Each repeat/week is indicated with a different line pattern (week one – solid line; week two ‘—’, week three ‘….’, week four ‘-.-.-.’). Each column represents one participant (N = 6). Pre: sample collected immediately pre-feeding; post: sample collected immediately post-feeding. **Figure S3.** Inter- and intra-individual variation in breastmilk cell viability (%) by study week. The top row shows the feeding breast, whereas the bottom row shows the non-feeding (control) breast. Each repeat/week is indicated with a different line pattern (week one – solid line; week two ‘—’, week three ‘….’, week four ‘-.-.-.’). Each column represents one participant (N = 6). Pre: sample collected immediately pre-feeding; post: sample collected immediately post-feeding. **Figure S4.** Inter- and intra-individual variation in breastmilk protein concentration (g/L) by study week. The top row shows the feeding breast, whereas the bottom row shows the non-feeding (control) breast. Each repeat/week is indicated with a different line pattern (week one – solid line; week two ‘—’, week three ‘….’, week four ‘-.-.-.’). Each column represents one participant (N = 6). Pre: sample collected immediately pre-feeding; post: sample collected immediately post-feeding. **Figure S5.** Inter- and intra-individual variation in breastmilk fat content by breast type. The top row shows the right breast, whereas the bottom row shows the left breast. Each column represents one participant (N = 6). Pre: sample collected immediately pre-feeding; post: sample collected immediately post-feeding. **Figure S6.** Inter- and intra-individual variation in breastmilk cell content (counts/mL milk) by breast type. The top row shows the right breast, whereas the bottom row shows the left breast. Each column represents one participant (N = 6). Pre: sample collected immediately pre-feeding; post: sample collected immediately post-feeding. **Figure S7.** Inter- and intra-individual variation in breastmilk cell viability (%) by breast type. The top row shows the right breast, whereas the bottom row shows the left breast. Each column represents one participant (N = 6). Pre: sample collected immediately pre-feeding; post: sample collected immediately post-feeding. **Figure S8.** Inter- and intra-individual variation in breastmilk protein content (g/L) by breast type. The top row shows the right breast, whereas the bottom row shows the left breast. Each column represents one participant (N = 6). Pre: sample collected immediately pre-feeding; post: sample collected immediately post-feeding. **Figure S9.** Inter- and intra-individual variation in breastmilk fat content by expression type. The top row shows the pump expressions, whereas the bottom row shows the hand expressions. Each column represents one participant (N = 6). Pre: sample collected immediately pre-feeding; post: sample collected immediately post-feeding. **Figure S10.** Inter- and intra-individual variation in breastmilk cell content (counts/mL milk) by expression type. The top row shows the pump expressions, whereas the bottom row shows the hand expressions. Each column represents one participant (N = 6). Pre: sample collected immediately pre-feeding; post: sample collected immediately post-feeding. **Figure S11.** Inter- and intra-individual variation in breastmilk cell viability (%) by expression type. The top row shows the pump expressions, whereas the bottom row shows the hand expressions. Each column represents one participant (N = 6). Pre: sample collected immediately pre-feeding; post: sample collected immediately post-feeding. **Figure S12.** Inter- and intra-individual variation in breastmilk protein content (g/L) by expression type. The top row shows the pump expressions, whereas the bottom row shows the hand expressions. Each column represents one participant (N = 6). Pre: sample collected immediately pre-feeding; post: sample collected immediately post-feeding.
(PDF)
