# Supplementary material for: Breastmilk Cell and Fat Contents Respond Similarly to Removal of Breastmilk by the Infant
Source: PLoS One. 2013 Nov 6;8(11):e78232. doi: 10.1371/journal.pone.0078232 (PMC3819380; doi:10.1371/journal.pone.0078232)
Supplement: File S1 — Inter- and intra-individual variation in breastmilk composition by study week, breast type (right or left), and expression type (pump or hand expression). File contains the following figures: Figure S1. Inter- and intra-individual variation in breastmilk fat content by study week. The top row shows the feeding breast, whereas the bottom row shows the non-feeding (control) breast. Each repeat/week is indicated with a different line pattern (week one – solid line; week two ‘—’, week three ‘….’, week four ‘-.-.-.’). Each column represents one participant (N = 6). Pre: sample collected immediately pre-feeding; post: sample collected immediately post-feeding. Figure S2. Inter- and intra-individual variation in breastmilk cell content (counts/mL milk) by study week. The top row shows the feeding breast, whereas the bottom row shows the non-feeding (control) breast. Each repeat/week is indicated with a different line pattern (week one – solid line; week two ‘—’, week three ‘….’, week four ‘-.-.-.’). Each column represents one participant (N = 6). Pre: sample collected immediately pre-feeding; post: sample collected immediately post-feeding. Figure S3. Inter- and intra-individual variation in breastmilk cell viability (%) by study week. The top row shows the feeding breast, whereas the bottom row shows the non-feeding (control) breast. Each repeat/week is indicated with a different line pattern (week one – solid line; week two ‘—’, week three ‘….’, week four ‘-.-.-.’). Each column represents one participant (N = 6). Pre: sample collected immediately pre-feeding; post: sample collected immediately post-feeding. Figure S4. Inter- and intra-individual variation in breastmilk protein concentration (g/L) by study week. The top row shows the feeding breast, whereas the bottom row shows the non-feeding (control) breast. Each repeat/week is indicated with a different line pattern (week one – solid line; week two ‘—’, week three ‘….’, week four ‘-.-.-.’). Each column represents one [file pone.0078232.s001.pdf]

SUPPORTING INFORMATION

Breastmilk cell and fat contents respond similarly to removal of breastmilk by the infant

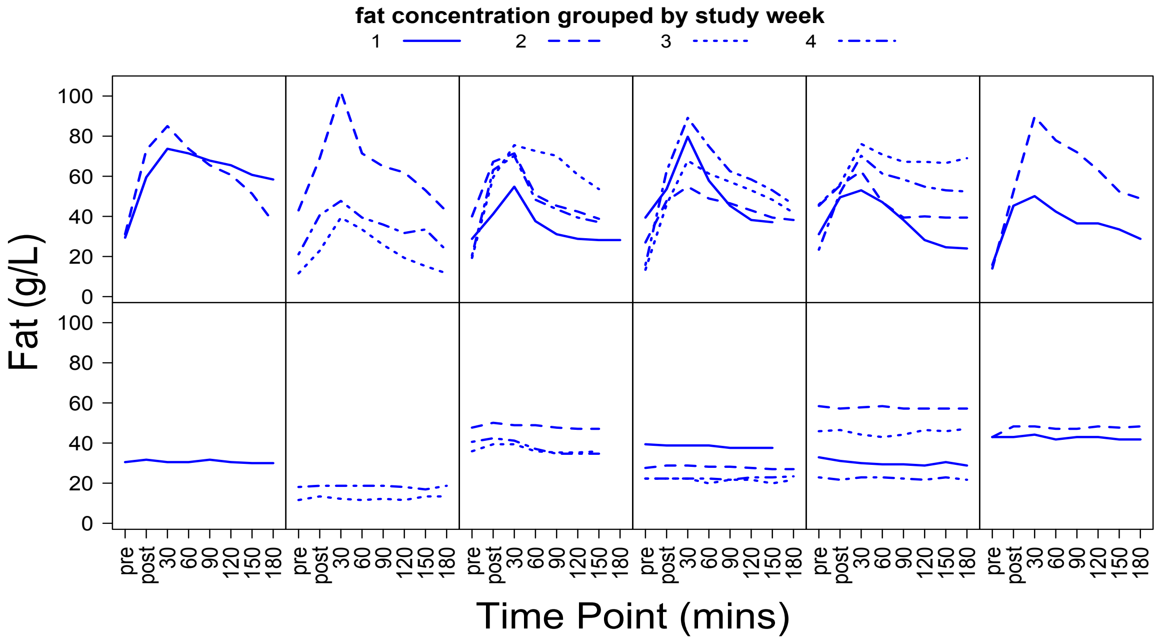

Figure S1. Inter- and intra-individual variation in breastmilk fat content by study week. The top row shows the feeding breast, whereas the bottom row shows the non-feeding (control) breast. Each repeat/week is indicated with a different line pattern (week one – solid line; week two '---', week three '….', week four '-.-.-.'). Each column represents one participant (N=6). Pre: sample collected immediately pre-feeding; post: sample collected immediately post-feeding.

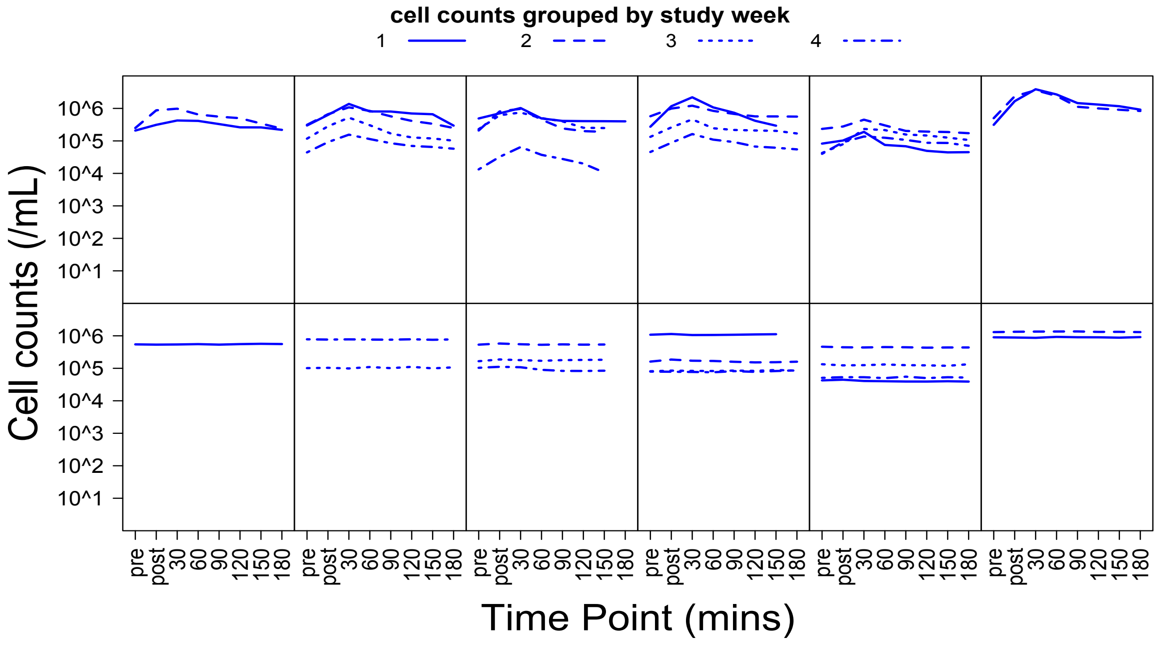

Figure S2. Inter- and intra-individual variation in breastmilk cell content (counts/mL milk) by study week. The top row shows the feeding breast, whereas the bottom row shows the non-feeding (control) breast. Each repeat/week is indicated with a different line pattern (week one – solid line; week two '---', week three '….', week four '-.-.-.'). Each column represents one participant (N=6). Pre: sample collected immediately pre-feeding; post: sample collected immediately post-feeding.

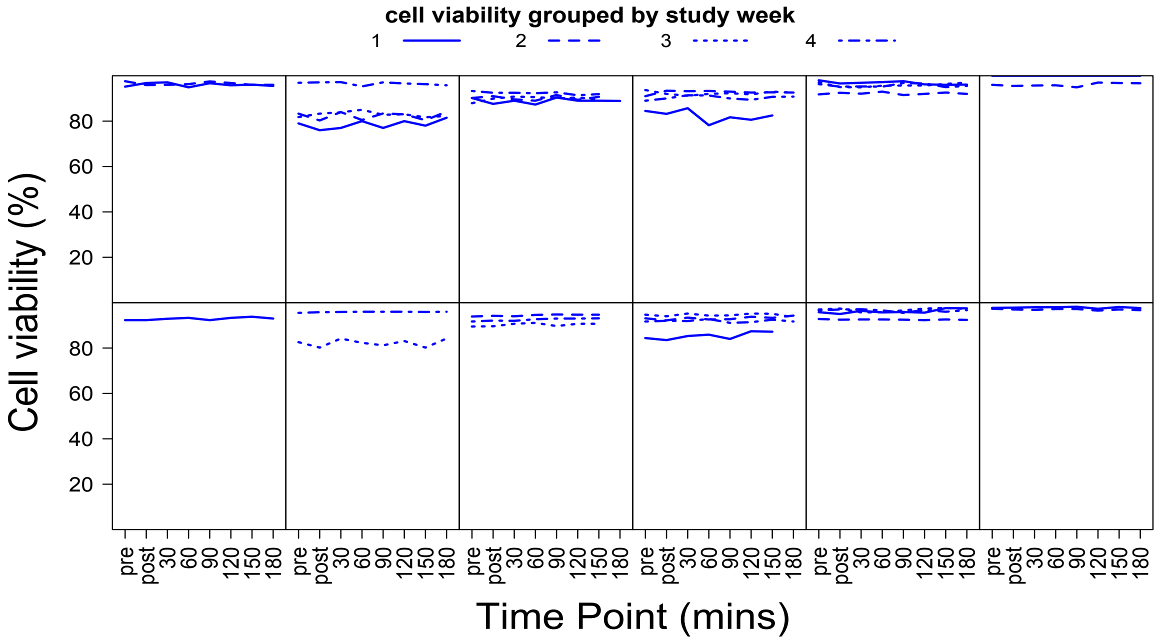

Figure S3. Inter- and intra-individual variation in breastmilk cell viability (%) by study week. The top row shows the feeding breast, whereas the bottom row shows the non-feeding (control) breast. Each repeat/week is indicated with a different line pattern (week one – solid line; week two '---', week three '….', week four '-.-.-.'). Each column represents one participant (N=6). Pre: sample collected immediately pre-feeding; post: sample collected immediately post-feeding.

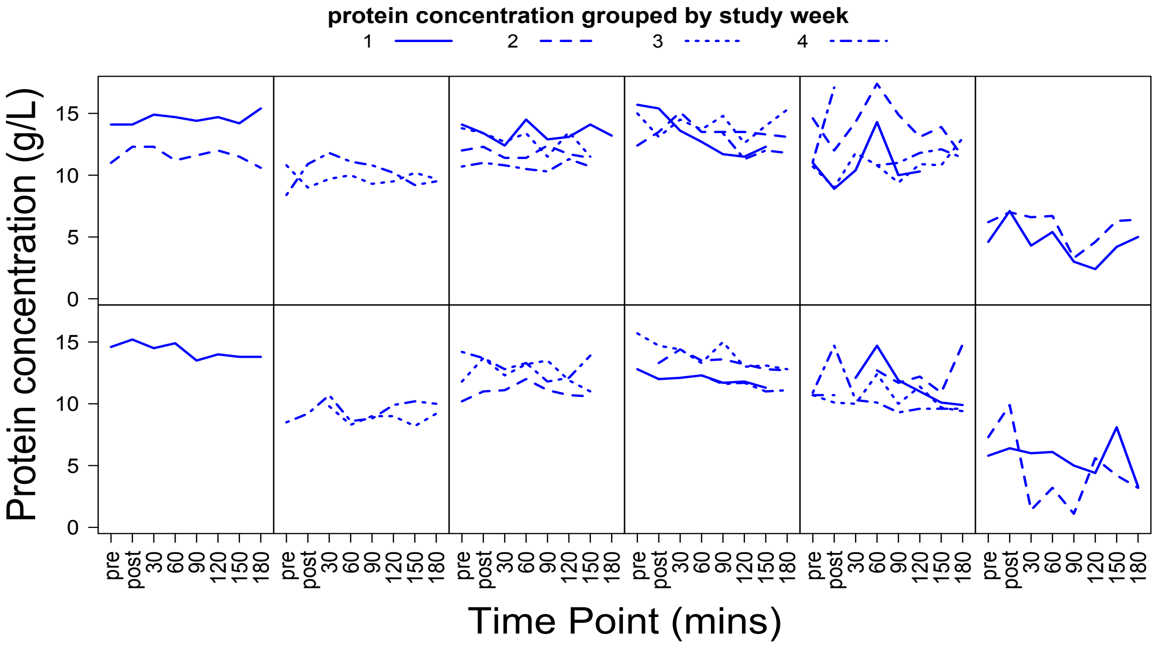

Figure S4. Inter- and intra-individual variation in breastmilk protein concentration (g/L) by study week. The top row shows the feeding breast, whereas the bottom row shows the non-feeding (control) breast. Each repeat/week is indicated with a different line pattern (week one – solid line; week two '---', week three '….', week four '-.-.-.'). Each column represents one participant (N=6). Pre: sample collected immediately pre-feeding; post: sample collected immediately post-feeding.

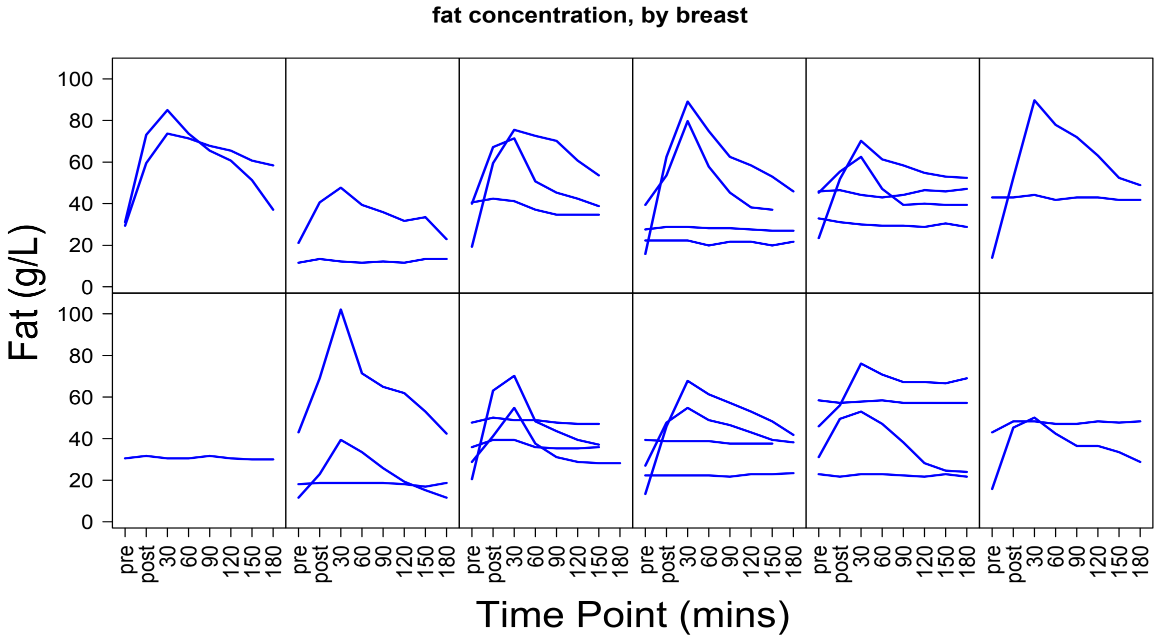

Figure S5. Inter- and intra-individual variation in breastmilk fat content by breast type. The top row shows the right breast, whereas the bottom row shows the left breast. Each column represents one participant (N=6). Pre: sample collected immediately pre-feeding; post: sample collected immediately post-feeding.

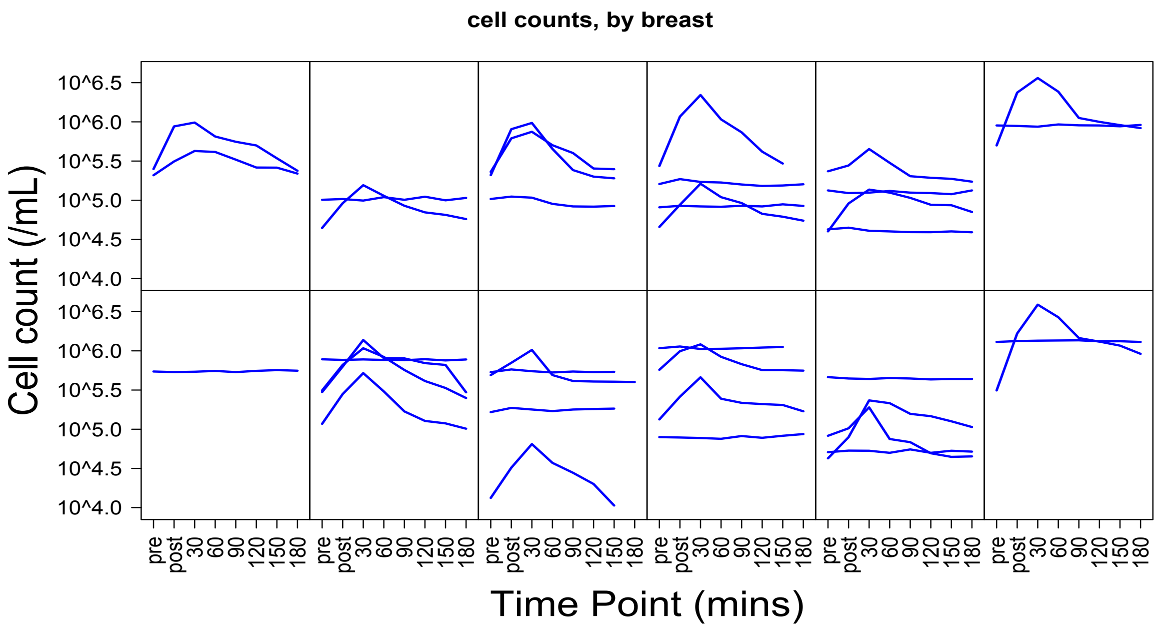

Figure S6. Inter- and intra-individual variation in breastmilk cell content (counts/mL milk) by breast type. The top row shows the right breast, whereas the bottom row shows the left breast. Each column represents one participant (N=6). Pre: sample collected immediately pre-feeding; post: sample collected immediately post-feeding.

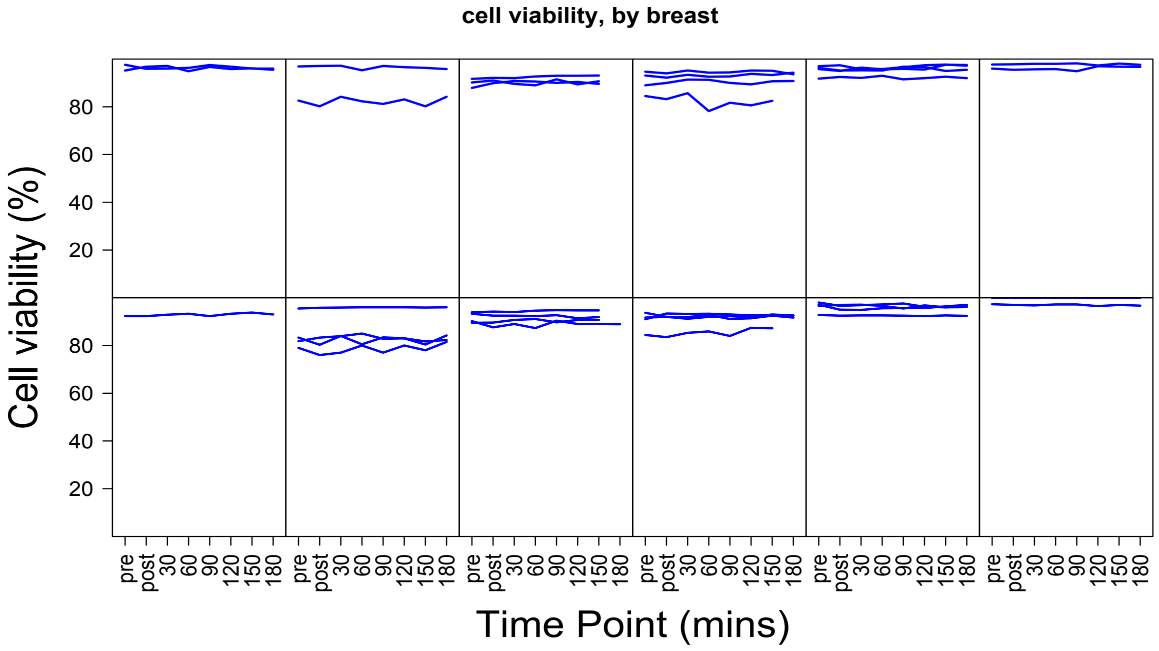

Figure S7. Inter- and intra-individual variation in breastmilk cell viability (%) by breast type. The top row shows the right breast, whereas the bottom row shows the left breast. Each column represents one participant (N=6). Pre: sample collected immediately pre-feeding; post: sample collected immediately post-feeding.

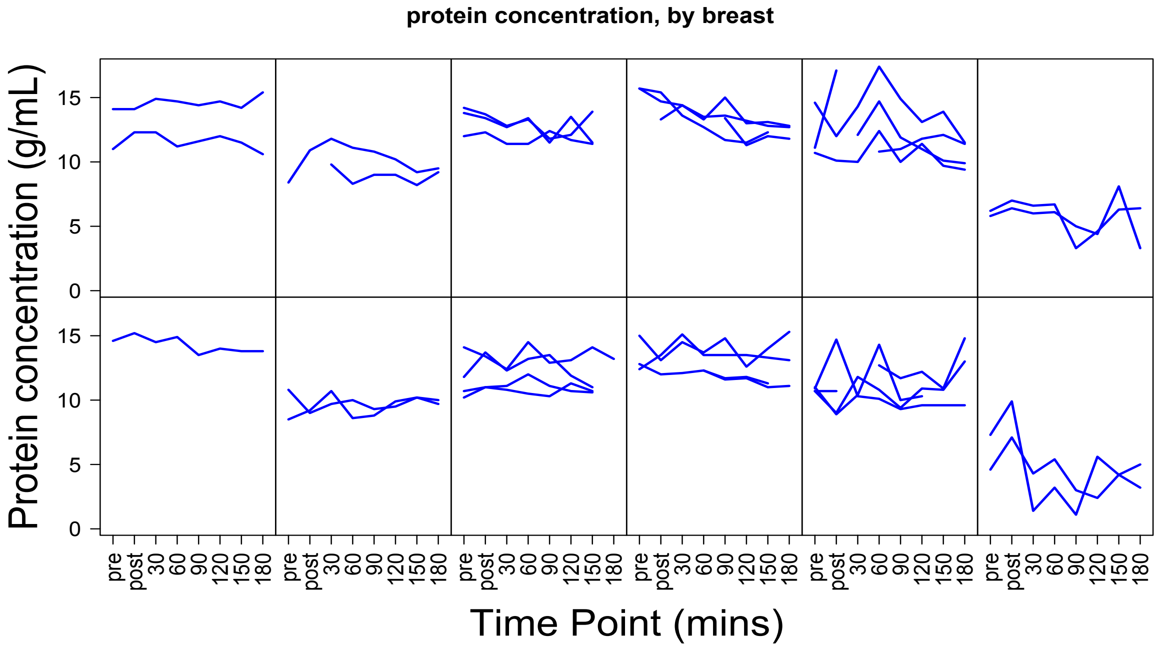

Figure S8. Inter- and intra-individual variation in breastmilk protein content (g/L) by breast type. The top row shows the right breast, whereas the bottom row shows the left breast. Each column represents one participant (N=6). Pre: sample collected immediately pre-feeding; post: sample collected immediately post-feeding.

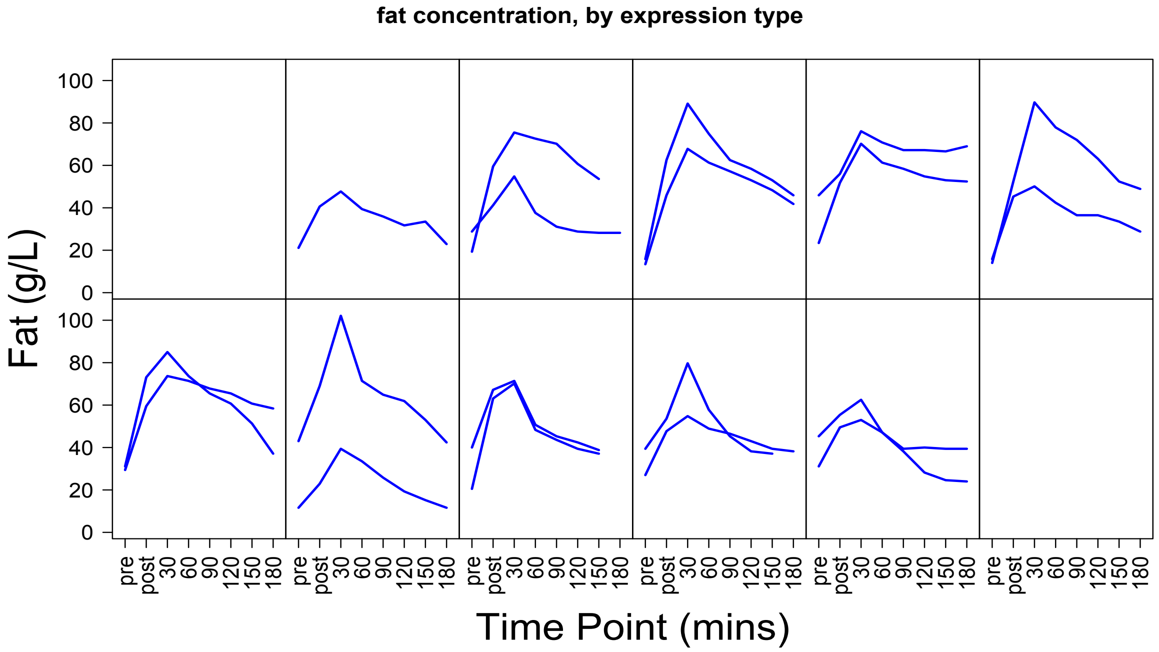

Figure S9. Inter- and intra-individual variation in breastmilk fat content by expression type. The top row shows the pump expressions, whereas the bottom row shows the hand expressions. Each column represents one participant (N=6). Pre: sample collected immediately pre-feeding; post: sample collected immediately post-feeding.

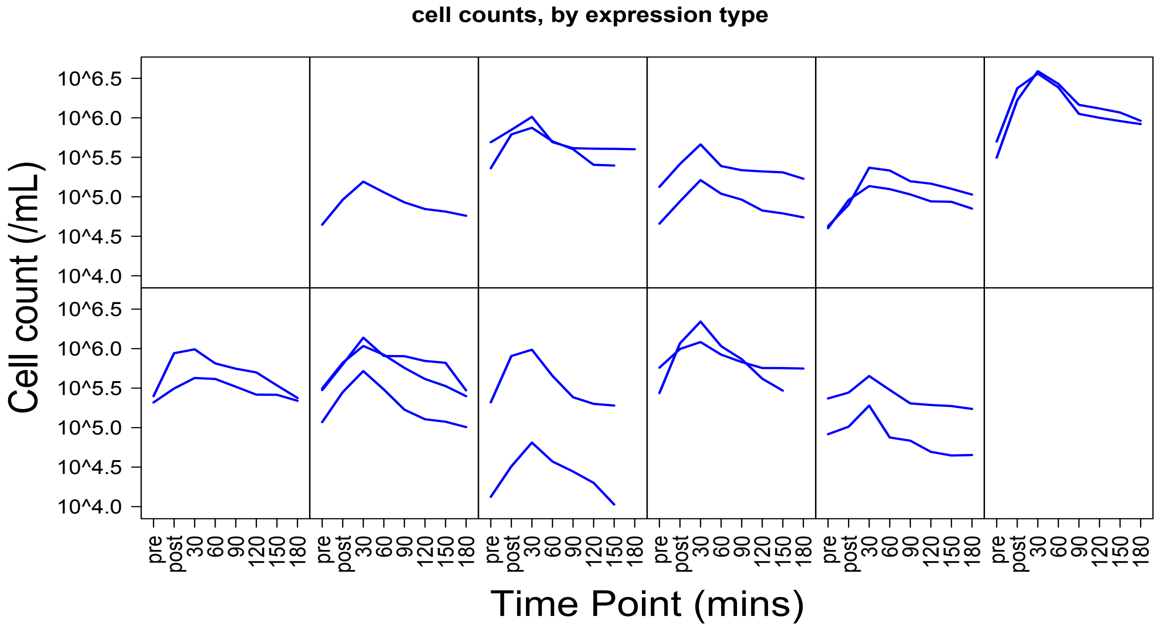

Figure S10. Inter- and intra-individual variation in breastmilk cell content (counts/mL milk) by expression type. The top row shows the pump expressions, whereas the bottom row shows the hand expressions. Each column represents one participant (N=6). Pre: sample collected immediately pre-feeding; post: sample collected immediately post-feeding.

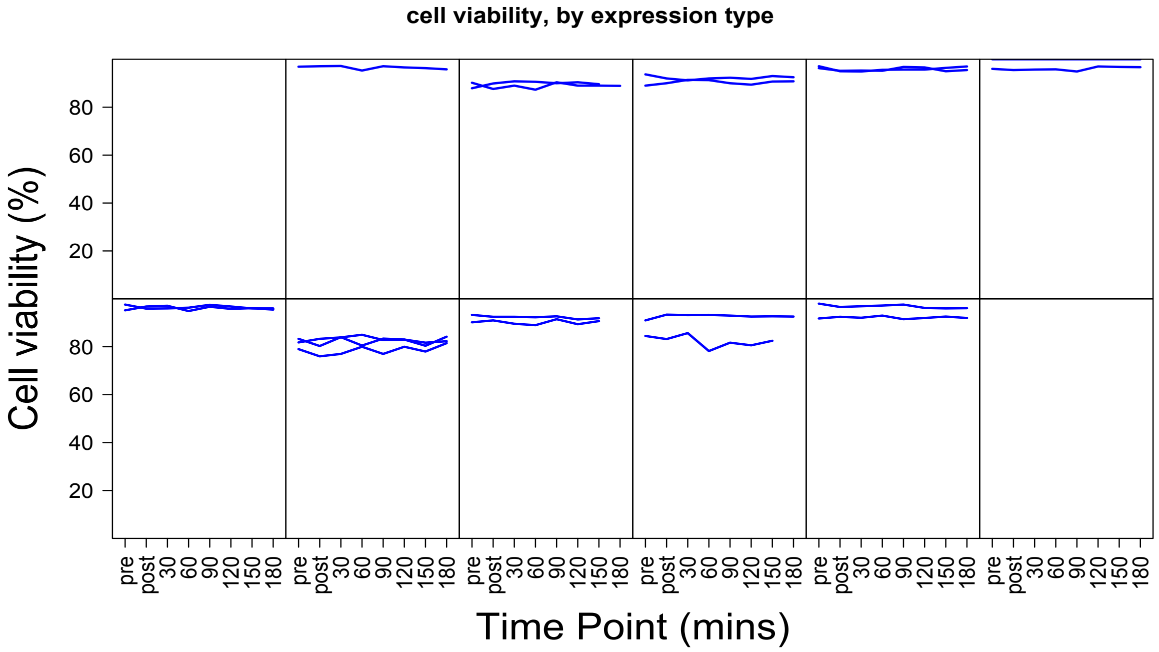

Figure S11. Inter- and intra-individual variation in breastmilk cell viability (%) by expression type. The top row shows the pump expressions, whereas the bottom row shows the hand expressions. Each column represents one participant (N=6). Pre: sample collected immediately pre-feeding; post: sample collected immediately post-feeding.

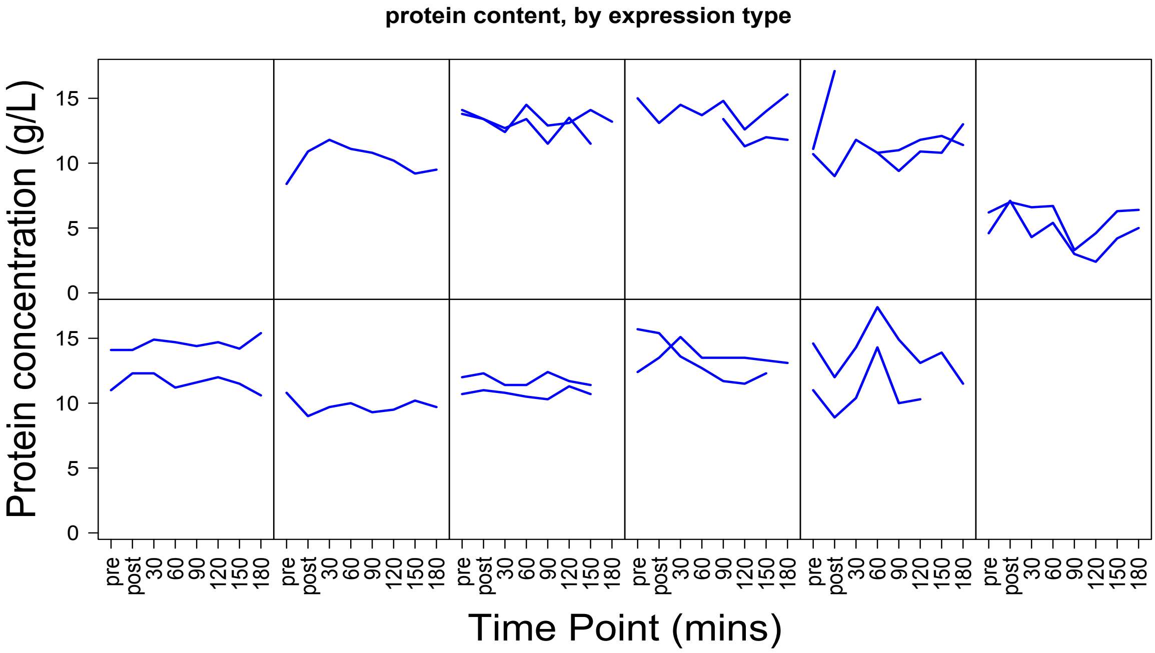

Figure S12. Inter- and intra-individual variation in breastmilk protein content (g/L) by expression type. The top row shows the pump expressions, whereas the bottom row shows the hand expressions. Each column represents one participant (N=6). Pre: sample collected immediately pre-feeding; post: sample collected immediately post-feeding.
